# Letter from Dr. S. Parsons

**Published:** 1848-01

**Authors:** S. Parsons, C. A. Harris

**Affiliations:** Baltimore.


### ARTICLE IV.

### *Letter from* Dr. S. Parsons.

Savannah, Dec. 5th, 1847.

Dear Sir:—In conformity with your request I give the following particulars of a case of galvanic action, and severe pain in the face, caused by two metals in the mouth.

Mrs. A. of a muco-bilious habit, (in good health,) called upon me to have some of her teeth filled. In filling the right superior first molar which was so much decayed in the centre of the grinding surface as to admit readily two sheets No. 6 gold foil, I took a large wedge-shaped plugger and forced it through the centre of the plug, forcing the gold firmly against the walls of the cavity. I then filled up this hole with more gold. Thinking I might get in more metal, I took a smaller wedge-shaped plugger, and forced it in as before, the gold had now become so hard that the point of the instrument broke off in the middle of the plug; it was so firmly imbedded in the gold that to remove it without removing the entire plug was impossible. As she had already become weary of sitting, (having filled a number,) and as she was in haste to return to her family, I closed the gold over the piece of the instrument, giving the plug as good a surface as if no portion of the instrument had remained. I informed her what I had done; expressed some fear that it would not do well, but assured her that I would re-fill it at any time if necessary. About three days after, her husband informed me that his lady suffered very much from pain on that side of the face; that they had made use of cataplasms, stimulants, &c. to no purpose, and wished to know what could be done. I immediately suggested to him that it was galvanic action, and that the removal of the plug, alone, would afford relief. She accordingly called on me again, I examined her mouth; there was no perceptible swelling of the surrounding parts, and but very little inflammation, yet the pain was severe, and the tooth a little sore to the touch. I removed the plug, and the pain ceased immediately. Two or three days after the soreness had left the tooth, I refilled it, and it has given no farther trouble.

I will simply add, this occurred in the latter part of August, 1847. Yours, &c.

S. PARSONS.

Dr. C. A. Harris, *Baltimore.*

